# The effect of psychoeducation regarding relaxation breathing, in stress reduction in a sample of nurses in a Greek hospital

**DOI:** 10.1192/j.eurpsy.2021.1922

**Published:** 2021-08-13

**Authors:** G. Lyrakos, D. Androutsos, E. Aslani, V. Spinaris

**Affiliations:** 1 Department Of Psychiatry, General Hospital of Nikaia “Ag. Panteleimon”, Nikaia, Greece; 2 2nd Department Of Pathology, General Hospital of Nikaia “Ag. Panteleimon”, Nikaia, Greece

**Keywords:** Psychoeducation, stress, relaxation techniques

## Abstract

**Introduction:**

Stress is one of the biggest problems leading a large portion of people to seek medical or psychotherapeutic management, while a large portion of hospital staff report high levels of work-related stress.

**Objectives:**

The purpose of this intervention was to implement a psychoeducation seminar on stress management by implementing diaphragmatic breathing exercises and to detect the reduction of its levels in nursing staff.

**Methods:**

The study took place at the General Hospital of Nikaia. Fifty employees, 38 women, aged 20-60 (M=37.4±10.5) participated in a two-hour group psychoeducation workshop, concerning psychoeducation on stress and application in diaphragmatic breathing exercises. The measurement of the success of the intervention was performed using a proportional stress assessment scale before and after the intervention. Statistical analysis was performed with SPSS26.

**Results:**

Stress levels before the intervention ranged from 0 to 10 (M=5.7±5.7) while after the intervention ranged from 0 to 7 (M=2.3±2.04). Age did not appear to play a role in stress reduction, but was found to be positively and significantly associated with pre-existing stress in employees (r=0.423 p=0.002). On the contrary, gender was found to be related both to the pre-existence of stress, with women reporting the highest levels (t=-3.534 p=0.001), and to the reduction of stress after the intervention (t=-2,534 p=0.001).

**Conclusions:**

The above findings indicate the importance of implementing group psychoeducation programs to reduce stress at the organizational level, a very important result considering the cumulative effect that the recent existence of covid-19 has had on nursing staff.

**Disclosure:**

No significant relationships.

